# Prediction of Antigenic Vaccine Peptide Candidates From BfmRS Associated With Biofilm Formation in Acinetobacter baumannii

**DOI:** 10.7759/cureus.47804

**Published:** 2023-10-27

**Authors:** A.S. Smiline Girija

**Affiliations:** 1 Department of Microbiology, Saveetha Dental College and Hospitals, Saveetha Institute of Medical and Technical Sciences [SIMATS] Saveetha University, Chennai, IND

**Keywords:** acinetobacter baumanii, in-silico, epitope peptides, bmfs, bmfr

## Abstract

Introduction: *A. baumannii* is categorized as a priority pathogen due to its propensity for multi-drug resistance, exhibiting resistance against the last resort of antibiotics. It is also considered a potent nosocomial pathogen, so targeting the microbe using novel strategies would be the need of the hour. In this context, the in-silico computational approach would serve the best to design the possible epitope peptides, which may be further considered for the experimental trials for their immunological response.

Objective: To predict the immune-dominant epitope peptide candidates against the bfmR and bfmS proteins mediating the two-component system adaptation in the formation of biofilm in *A. baumannii*.

Materials and methods: 11 different FASTA sequences of bfmR and bfmS from *A. baumannii* strains retrieved based on the blast-p similarity search tool were subjected to linear epitope B-cell epitope predictions under the IEDB B-cell epitope prediction server. Further analysis on antigenicity, allergenicity, and toxigenicity was achieved using the AntigenPro, Vaxijen, and AlgPred tools, with the physical and chemical properties evaluated using the Expasy Protparam server. Selection of the immunodominant peptides for T-cells was done through the databases under IEDB. The final assessment of protein-TLR2 interactions was done by MHC cluster servers.

Results: Four peptide sequences (E1-E4) were predicted for B-cell dominance, with E1, E2, and E4 as probable antigens. All were soluble and non-toxigenic. E1 and E3 were considered non-allergens. GRAVY values were negative for all the peptides, indicating the protein to be hydrophilic in nature. Analysis of the T-cell epitopes was promising, with 100% conservancy for class-I HLA alleles, high interaction scores for similarity with TLR2, and more hydrogen bonds for E2, followed by other epitope peptides.

Conclusion: The promising four epitopes, as predicted for bfmR and bfmS in the present study, suggest their potent role as possible candidates for the design of vaccines targeting the TCS of *A. baumannii*, recommending further in vitro and in vivo experimental validation.

## Introduction

Two-component system-associated virulence in *Acinetobacter bauamannii* contributes to its survival in varying harsh environments, making it a potent pathogen in hospital settings. *A. baumannii* was thus considered a priority pathogen by WHO (WHO, 2017), and it falls under the ESKAPE group [1]. It is known to induce various recalcitrant infections in hospitalized patients, exhibiting its exorbitance in the mortality rate [2] due to its multi-drug and extensive drug resistance properties [3]. With the observation of resistance against different classes of antibiotics and their prevalence in our earlier reports [4], The major transformation of this low-priority pathogen as a predominant pathogen is often associated with its two-component system (TCS's)-mediated adaptations. TCS seems to be a sensible system in *A. baumannii*, helps them to survive in varying atmospheric parameters, aids in modifying and regulating the expression of various metabolic pathways, and encompasses nearly 20 operons/genes [5].

bfmRS is a fascinating operon that is associated with the biofilm formation initiated by the attachment of pili, mediated by the csu operon, and documented to be regulated by the bfmRS gene [6]. Capsule formation is also prominent, with *A. baumannii* playing a vital role in adhesion and biofilm formation. Studies document the increased expression of the K-loci inducing the formation of capsules, which is mediated by the bmfRS operon [7]. The role of bfmRS in regulating exopolysaccharide production is also noteworthy in understanding its protective role against serum, enhancing its virulence in animal models [8]. Gene expression studies on bfmRS have shown an interesting finding: the promoter has a higher affinity for the inactive dephosphorylated structure than the activated phosphorylated structure upon analysis of its crystal structures [9]. 

Based on the sequential studies associated with bfmRS, it is evident that it could be an excellent drug target for the control of *A. baumannii* infections. With the design and evaluation of the vaccine peptides being considered as an alternative strategy, the present investigation intends to predict potent epitopes from bfmR and bfmS based on an immuno-informatic approach. This investigation would unravel the promiscuous immunogenic, non-toxigenic, and non-allergenic peptides from bfmRS that could lead to the synthesis of newer vaccine candidates against the TCS component of *A. baumannii*, arresting biofilm formation. The aim of the study was thus framed to identify the putative epitope peptides from the bfmR and bfmS of *A. baumannii* and to evaluate their immunological properties using bioinformatics tools and databases. 

## Materials and methods

Selection of bfmR and bfmS protein from *A. baumannii*

Using the UNIPROT database (https://www.uniprot.org/), FASTA sequences of bfmR (ID Q2VSW6) and bmfS (ID Q2VSW5) proteins were retrieved from the clinical isolates of *A. baumannii* and were subjected to blast-p analysis for similar proteins for a further selection of amino-acid sequences.

Predictions of B-cell dominant epitopes

Sequences of bfmR and bfmS from FASTA were subjected to the predictions of the linear B-cell epitopes as an input in the predictions for B-cell interactions in the database under IEDB (http://tools.iedb.org/main/bcell/). Identification of the putative epitopes was based on the collection of sequential methods for characterizing the epitopes from the amino-acid input sequences.

Antigenicity predictions

To assess the antigenic properties of the identified epitopes from bfmR and bfmS, an alignment-independent categorization based on their physico-chemical properties was achieved through the VaxiJen v2.0 server [10]. It renders an output statement with a predefined cut-off value, with the precision of the antigenicity prediction varying from 70%-80%. In addition, for the predicted peptides, a sequence-based alignment-free assessment for antigenicity was achieved by the ANTIGENpro server [11,12]. 

Physical and chemical properties of the predicted epitopes

Predicted peptides from bfmR and bfmS were further evaluated for their molecular weight, theoretical pI, composition of amino acids and atoms, extinction coefficient, estimated half-life, instability index, aliphatic index, and grand average of hydropathicity (GRAVY) using the computational approach in the ProtParam Expasy server [13].

Allergenicity and toxigenicity predictions

For further predictions of the bfmR and bfmS peptides for their allergenic and toxigenic properties, we applied a systematic approach with high accuracy in AlgPred software [14]. The similarity between the epitopes was evaluated based on the default parameters set in the AlgPred tool. Evaluation of the toxigenicity was achieved by the toxinpred tool [15], where predictions based on the parameters of hydrophobicity, hydrophylicity, and hydropathicity were interpreted based on the SVM score and charge.

Signal location predictions

Localization of the peptides being the crucial step in vaccine design and drug delivery; it was achieved using the neural network of two transmembrane sets to verify the signals and locations of the peptides bfmR and bfmS using SignalP 4.1 [16]. Using SignalP 5.0, discrimination of the proteins into three different categories, viz., secretory signal peptides, lipoprotein signal peptides, and tat signal peptides, was achieved.

Predictions on continuous B-cell epitopes

Immune Epitope Database and Analysis (IEDB server) encompasses prediction tools for B-cell continuous epitopes, viz., BepiPred 2.0 sequential epitope predictions, flexibility predictions (Karplus-Schulz), beta turn predictions (Chou-Fasman), antigenicity predictions (Kolaskar Tongaonkar), surface accessibility predictions (Emini), and hydrophilicity predictions (Parker) [17-21]. These approaches were applied to the peptide sequences of bfmR and bfmS that analyze the peptides based on the set default parameters. On a propensity-based scale measurement, the output is interpreted based on the X axis (residue positions) and Y axis (residue scores) as the yellow portion on the graphs, interpreted as epitopes of higher probability.

Predictions on T-cell epitopes and MHC class allelles

A consensus method of approach was applied to predict the T-cell dominant antigenic epitopes from bfmR and bfmS that could be recognized by the T-cells and MHC class I binding. The calculation and selection were based on a decreasing order, viz., Consensus > ANN > SMM > NetMHCpan EL > CombLib, based on the frequent alleles with at least 1% of the human population or higher frequencies of alleles. The selection was further based on the percentile ranks and the binding affinities based on the IC50 values. 

Conservancy and class I immunogenicity analysis

Assessment of the potent immunogens from bfmR and bfmS proteins was achieved with the set parameters (IEDB server), and the epitopes possessing positive scores were selected as the best epitope candidates. In addition, the predicted epitope’s conservancy with the protein that was selected was evaluated by the IEDB conservancy analysis server.

Cluster analysis for the MHC restricted alleles

Functional assessment of the interaction of the selected peptides with HLA alleles on MHC was deduced (MHC cluster v2.0 server). The output was interpreted by assessing the static heat map and the graphical tree obtained between the clusters and peptides.

Protein-TLR-2 receptor interactions

In vaccine design, the structure optimization is very essential. This was assessed based on the protein-peptide binding and the TLR2 interactions of the epitopes predicted (Galaxy web server). The output was obtained in terms of interaction similarity scores and the number of hydrogen bonds. 

## Results

Blast-p similar protein selection

Retrieved FASTA sequences of bfmR (ID Q2VSW6) and bfmS (ID Q2VSW5) upon blast-p similarity search yielded 9 proteins (Accession No: WP_000076440.1, WP_119523011.1, OFD29399.1, OWX87057.1, WP_113755212.1, WP_141102339.1, 5HM6_A Chain A, 5E3J_A Chain A & RKL57993.1) and two proteins (Accession No. WP_000472465.1 and WP_050499517.1) for bfmR and bfmS, respectively, with the description as DNA binding response regulator and sensor histidine kinase.

Determination of B-cell epitopes

Peptide mapping of the 11 FASTA sequences of bfmR and bfmS from various strains of A. baumannii (IEDB B-cell epitope prediction of linear epitopes) yielded 12 epitopes (default threshold set->0.5) with the four common epitope sequences (E1-13aa, E2-10aa, E3-12aa, and E4-10aa) for further analysis (Table 1).

**Table 1:** Predictions of B-cell linear epitopes from bmfR and bmfS of A. baumannii

Target protein	Accession No.	Description of function and strain	Start	End	Similar peptide	Epitope designated	Peptide length
bmfR	WP_000076440.1	MULTISPECIES: response regulator transcription factor BfmR [Acinetobacter]	209	221	PKIGDDPENPKRI	E1	13
	WP_119523011.1	response regulator transcription factor BfmR, partial [Acinetobacter baumannii]	194	206	PKIGDDPENPKRI	E1	13
	OFD29399.1	DNA-binding response regulator, partial [Acinetobacter baumannii]	169	181	PKIGDDPENPKRI	E1	13
	OWX87057.1	DNA-binding response regulator, partial [Acinetobacter baumannii]	185	197	PKIGDDPENPKRI	E1	13
	WP_113755212.1	response regulator transcription factor BfmR, partial [Acinetobacter baumannii]	140	152	PKIGDDPENPKRI	E1	13
	WP_141102339.1	response regulator transcription factor BfmR, partial [Acinetobacter baumannii]	-	-	-	-	-
	5HM6_A Chain A	BfmR [Acinetobacter baumannii]	-	-	-	-	-
	5E3J_A Chain A	Response Regulator Rsta [Acinetobacter baumannii AB307-0294]	-	-	-	-	-
	RKL57993.1	DNA-binding response regulator, partial [Acinetobacter baumannii]	84	96	PKIGDDPENPKRI	E1	13
bmfS	WP_000472465.1	sensor histidine kinase BfmS [Acinetobacter baumannii]	56	65	VARQPGKQQK	E2	10
			465	476	DDGPGIPEQDRK	E3	12
			514	523	IKVDESPSLG	E4	10
	WP_050499517.1	sensor histidine kinase BfmS, partial [Acinetobacter baumannii]	56	65	VARQPGKQQK	E2	10
			465	476	DDGPGIPEQDRK	E3	12
			514	523	IKVDESPSLG	E4	10

**Figure 1:**
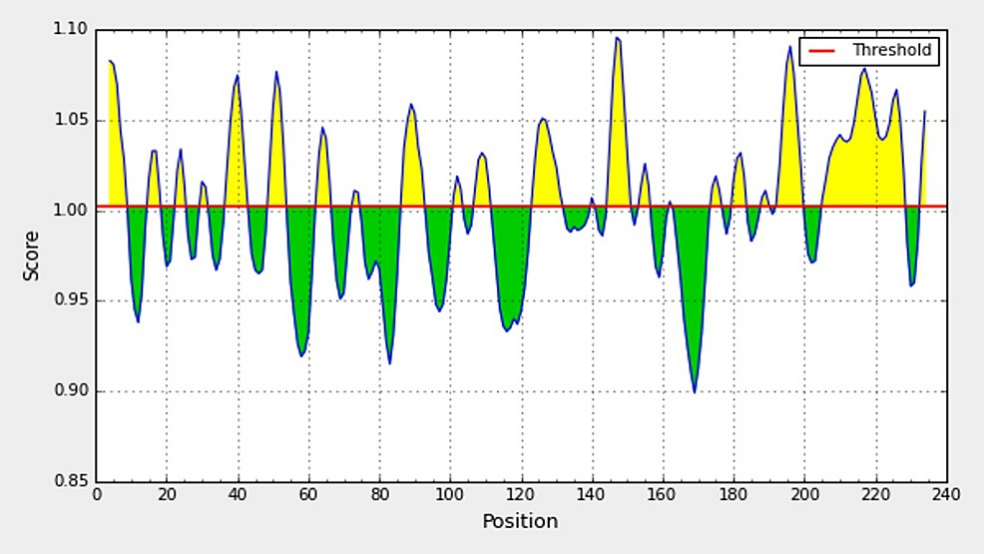
B-cell antigenic epitope predictions from bmfR and bmfS of A. baumannii, with the start and end positions showing the antigenic peptide sequences (as a yellow color peak)

The promiscuous antigenic positions on the graphical peaks of yellow color were interpreted with the values on the X and Y axes based on the biochemical properties of amino-acid composition, hydrophobicity, hydrophilicity, surface accessibility, and flexibility (Figure 1). 

Vaccine properties

Antigenic Potentials

AntigenPro software analysis of the epitopes showed E1, E2, and E4 as antigens except E4, and all four epitopes scored as soluble proteins (SolPro analysis: at a set threshold of ≥0.5) (Table 2).

**Table 2:** Antigenicity and solubility analysis of the predicted bmfR and bmfS epitopes by VaxiJen, ANTIGENPro, and SOLPro tools

Peptide	Epitope designations	Peptide sequence	VaxiJen		Antigen PRO	SolPro	
			Threshold value (≥0.4)			Threshold value (≥0.5)	
1.	E1	PKIGDDPENPKRI	0.6019	Antigen	0.404172	0.941907	Soluble
2.	E2	VARQPGKQQK	0.8272	Antigen	0.051816	0.895812	Soluble
3.	E3	DDGPGIPEQDRK	-0.0829	Non-Antigen	0.792621	0.997098	Soluble
4.	E4	IKVDESPSLG	1.0185	Antigen	0.602155	0.818919	Soluble

VaxiJen server 4.0 (threshold of >0.4) showed E3 with a negative value and all others with positive scores, with E1 as a potential vaccine candidate with the highest score of 1.0185. 

Allergenic and toxigenic properties

SVMc + IgE epitope + ARPsBLAST + MAST based-hybrid approach prediction on the allergenic properties showed E1 and E3 as asnon-allergens (Table 3).

**Table 3:** Allergenicity predictions of the baeR epitopes based on SVM and hybrid approaches (threshold value 0.4) by the AlgPred tool

Peptide	Predicted antigens	IgE	MAST	SVM-Aa	SVM-dp	BLAST - ARP	Hybrid
E1	PKIGDDPENPKRI	NA	NA	A	A	NA	NA/A
E2	VARQPGKQQK	-	-	-	-	-	-
E3	DDGPGIPEQDRK	NA	NA	A	A	NA	NA/A
E4	IKVDESPSLG	-	-	-	-	-	-

However, the allergenic properties were not predicted for E2 and E4, as the protein sequences of the peptides were smaller and could not be used as input sequences. SVM-AA and DP predicted both E1 and E3 as allergens. All the epitopes were observed as non-toxins under the Toxinpred tool with negative SVM scores, and the further parameters of hydropilicity, hydropathicity, and hydrophobicity were recorded (Table 4).

**Table 4:** Toxigenicity predictions of the bmfR and bmfS epitopes based on the ToxinPred tool

Peptide	Predicted antigens	SVM score	Prediction	Hydrophobicity	Hydropathicity	Hydrophilicity	Charge
E1	PKIGDDPENPKRI	-0.66	Non-toxin	-0.40	-1.73	1.12	0.00
E2	VARQPGKQQK	-1.12	Non-Toxin	-0.52	-1.88	0.76	3.00
E3	DDGPGIPEQDRK	-1.22	Non-Toxin	-0.45	-2.12	1.37	-2.00
E4	IKVDESPSLG	-0.91	Non-Toxin	-0.11	-0.20	0.45	-1.00

Physical and chemical properties of the peptides

ProtParam assessments on the physical and chemical properties of the peptides showed E1 and E3 as stable proteins with a shelf life of 20 hours and 1.1 hours, respectively (in vitro). GRAVY values scored negative values for all the peptides, suggesting their hydrophylicity, and E4 had the highest aliphatic scores (Table 5).

**Table 5:** Physico-chemical properties of the predicted bmfR and bmfS epitopes, viz., molecular weight (MW), iso-electric point (IP), stability index (SI), shelf life (SL), aliphatic index (AI) and grand average hydrophathicity (GRAVY)

Peptide	Predicted antigens	MW	IP	SI	SL	AI	GRAVY
E1	PKIGDDPENPKRI	1478.67	6.54	38.93 (stable)	>20 hours	60.00	-1.731
E2	VARQPGKQQK	1139.32	11.17	81.97 (Unstable)	100 hours	39.00	-1.880
E3	DDGPGIPEQDRK	1326.39	4.23	39.99 (stable)	1.1 hours	32.50	-2.117
E4	IKVDESPSLG	1044.17	4.37	59.45 (Unstable)	20 hours	107.00	-0.200

In par with constant equilibrium points and taking zero as the total liquid charge of the protein, the ProtParam server analyzed the iso-electric points, with E2 scoring the highest value with a pH of 6.48.

Analysis on the peptide signals

**Figure 2:**
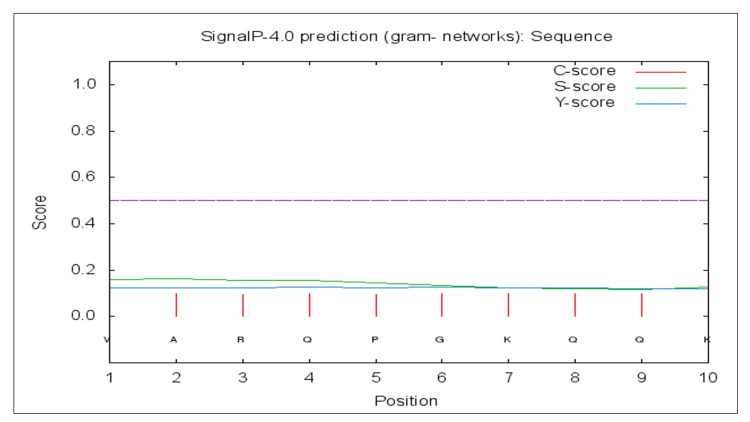
Signal P-noTM neural predictions based on the D-cut-off value using the Signal P 4.0 server [C score, S score, and Y score are depicted as pink, green, and blue, respectively]

Neural network analysis for transmembrane proteins achieved based on Signal P-4.1 did not show any transmembrane proteins. However, the likelihood of the proteins was good for the secretary signals, TAT signals, and lipoprotein signals under signal 5.0 server. The location of the epitope predicted based on the tool was achieved for two epitopes (E1 and E2), and both epitopes did not show transmembrane signals (D-cutoff value - 0.51 under signal-TM neural network) (Table 6, Figure 2). 

**Table 6:** SignalP 4.0 and 5.0 predictions for the bfmR and bfmS transmembrane peptides [D-cut off signal-noTM networks: 0.57, D-cut off signal-TM networks: 0.51]

Peptide	Predicted antigens	Signal 4.0 predictions		Signal 5.0 predictions			
		Score	Signal TM	Signal peptide (Sec/SPI)	TAT signal peptide (Tat/SPI)	Lipoprotein signal peptide (Sec/SPII)	Other
E1	PKIGDDPENPKRI	0.127	No	0.1671	0.1416	0.1277	0.5636
E2	VARQPGKQQK	0.139	No	0.157	0.1465	0.1415	0.5551
E3	DDGPGIPEQDRK	0.114	No	0.123	0.1131	0.0895	0.6744
E4	IKVDESPSLG	0.109	No	0.1737	0.1417	0.1646	0.52

T cell immune-dominant epitope selection

The consensus combinatorial score in the IEDB-AR server predicted eight T-cell dominant epitopes (9-10aa) assessed for their binding with MHC class I alleles based on the percentile ranks (≤ 0.2) eliciting higher affinity under ANN and SMM-based IC50 values (IC50 < 200 nm) (Table 7).

**Table 7:** Consensus method predictions with HLA alleles for T-cell MHC class-I binding epitopes based on the percentile ranks (lowest rank) and IC50 values (<50nM – high binding affinity)

Peptide sequences	Alleles	Start	End	Length	Peptide	Percentile rank	ANN IC_50_	ANN rank	SMM IC_50_	SMM rank	Comb Lib score	Comb Lib rank	Net MHC pan score	Net MHC pan rank
PKIGDDP ENPKRI	HLA-A*11:01	2	11	10	KIGDDPENPK	2.25	509.56	2.3	246.52	2.2	-	-	-	-
	HLA-A*03:01	2	11	10	KIGDDPENPK	3.35	990.6	2.9	701.78	3.8	-	-	-	-
VARQPGKQQK	HLA-A*11:01	1	10	10	VARQPGKQQK	6.95	2777.23	5.6	2817.41	8.3	-	-	-	-
	HLA-B*07:02	1	9	9	VARQPGKQQ	8.3	9597.04	8.3	4919.15	9.1	0.0002	6.5	-	-
DDGPGIPEQDRK	HLA-A*33:01	2	11	10	DGPGIPEQDR	7.3	8179.9	8.6	1669.17	6	-	-	-	-
	HLA-A*68:01	2	11	10	DGPGIPEQDR	10.45	6931.85	8.9	2182.68	12	-	-	-	-
IKVDESPSLG	HLA-B*40:01	1	9	9	IKVDESPSL	12.8	13402.63	8.6	16745.57	17	-	-	-	-
	HLA-A*02:06	1	9	9	IKVDESPSL	14	4890.62	13	733.75	15	-	-	-	-

The frequent interaction of HLA alleles with the class I T-cell immunodominant peptides was observed. Class I molecules showed 100% conservancy, with E1 and E3 peptides yielding a positive score of 0.0387 and 0.18906, respectively.

MHC restrictions and cluster analysis

**Figure 3:**
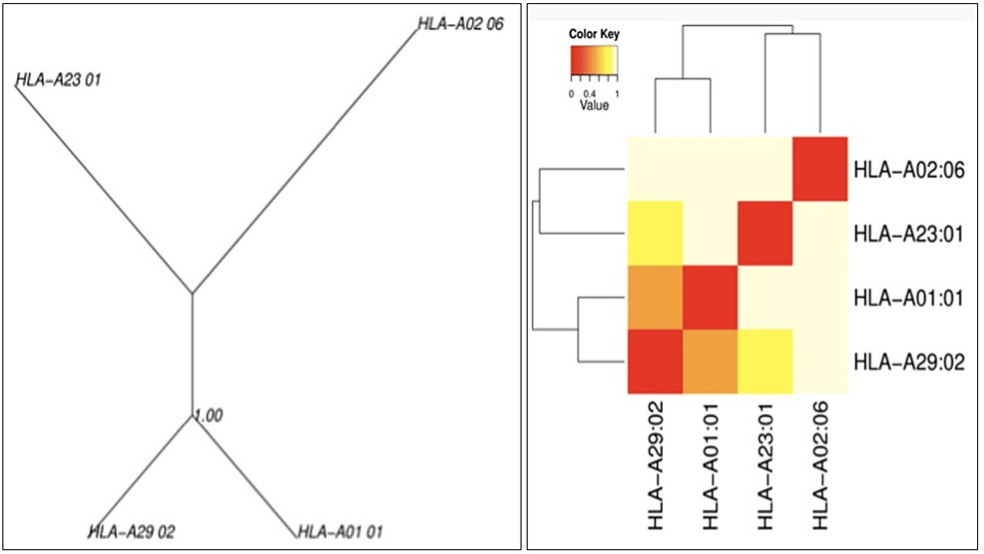
Cluster analysis representing the functional relationships between the predicted peptide E2 and HLA-class I alleles is represented by graphical tree and heat map formats (the red zone indicates strong interactions and the yellow zone indicates weak interactions)

The heat maps and the dynamic graphical tree for the finally selected E2 epitope peptide, as re-assessed by MHC cluster analysis, indicated strong (red) and weak (yellow) interactions with appropriate annotations for the HLA alleles (Figure 3).

Protein-peptide interactions

**Figure 4:**
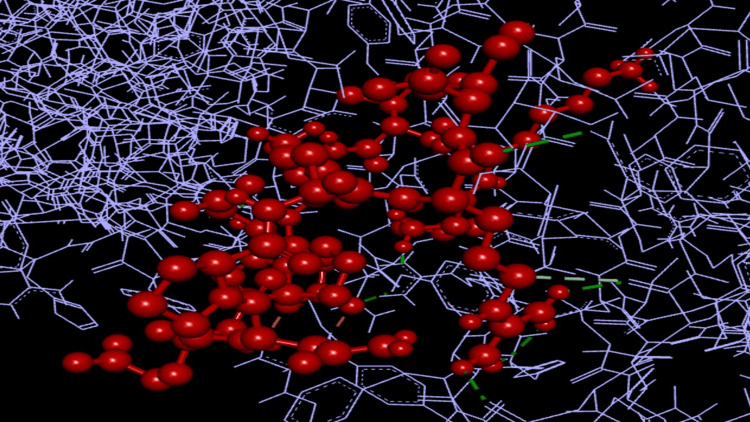
Protein-peptide interaction pictures of the predicted epitope E2 with the TLR-4 receptor (Red showing the ball and stick model of the epitope peptides and purple showing the line model of the TLR-4 receptor)

The amino-acid similarity of the peptides predicted (E1-E4: target complexes) aligned to the template structure (TLR-2) of the amino acids was assessed by the Galaxy web server. E2 scored as the best interacted peptide, with the highest number of 11 hydrogen bonds possessing -21 Kcal/mol as the interaction similarity score (Figure 4). E1, E3, and E4 showed eight hydrogen bonds, with the scores for the similarity of interaction being -19 kcal/mol, -16 kcal/mol, and -12 kcal/mol, respectively.

## Discussion

*A. baumannii* is considered an important opportunistic nosocomial pathogen, and the BfmRS plays a vital role in the two-component system, conferring both virulence and antimicrobial resistance. Its localization as an outer membrane protein contributes to the formation of the outer membrane vesicles, modulating the host immune response and cytotoxicity. Based on the varying stimulus from the environment, the TCR sensor kinase regulates and relays certain signals into the cytoplasm, followed by the BfmR regulating the expression of the csuA operon to form pili and later progressing into biofilm formation [22]. In this context, the study focuses on predicting immune-dominant epitope peptides within the bfmR and bfmS proteins of *A. baumannii*, an important nosocomial pathogen known for its multi-drug resistance. The research aims to identify potential epitopes that can be used in vaccine development to target the two-component system (TCS) adaptation of *A. baumannii*, specifically its role in biofilm formation. This study also suggests the possibility of promising vaccine design and evaluation using bfmR and bfmS as potent targets to combat the TCS-mediated adaptations in *A. baumannii*.

Signal transduction mechanisms play a vital role in bacterial adaptation; the role of TCS is considered one of the ubiquitous systems in many potent pathogens, including *A. baumannii*. This adaptation response in *A. baumannii* is vital to overcome the harsh hospital environments, transforming itself into a potent nosocomial pathogen and modulating its antibiotic susceptibility patterns and virulence modules. bfmR and bfmS were thus considered for the present study as they play a crucial role in regulating biofilm formation. The selection of the proteins under the UNIPROT database yielded just one protein under each category, and the blast similarity search of the FASTA sequences showed nine similar proteins for bfmR and two similar proteins for bfmS, and the similarity was 100% with fewer e-values amidst a total of 182 proteins reported from *A. baumannii*. Thus, we proceeded with the 11 proteins for further analysis. The common epitope sequence, as predicted under IEDB B-cell linear epitope predictions, designated as E1-E4, was utilized for the prediction analysis. It is astonishing to note the accessibility of the IEDB B-cell epitope tool, where we have retrieved the Figure 1 graphs with antigenic yellow epitope peaks based on various parameters and computed them with ease. 

According to the findings on the predictions for antigenicity under the ANTIGENPro and VaxiJen servers, under the set default parameters, it was possible to compare the threshold values to identify three putative antigenic peptides. SolPro predictions on solubility showing soluble peptides for all the predicted peptides is yet another good finding of the study. Predictions on allergenicity were not promising as we could not proceed with the analysis of E2 and E4 due to the low number of amino-acid sequences, which can also be considered a limitation of the server. However, the observation of non-allergens for E1 and E3 was a noteworthy finding, together with the non-toxic nature of the predicted peptides under the ToxinPred tool. 

The physico-chemical properties of the peptides were considered to be an important fact for the peptide synthesis for in-vitro vaccine trials, and they were efficiently deduced in the present study using the Expasy Protparam server. It was so fortunate to observe that the two predicted antigenic peptides under the ANTIGENPro and VaxiJen servers are stable proteins, with E1 possessing a shelf life of 20 hours. However, though E4 showed a similar shelf life and E4 had a 100-hour shelf life, they were predicted to be unstable. All the proteins were hydrophilic proteins by considering the GRAVY values predicted based on the formula using the added values of the hydropathy scores and the number of amino acid residues. The high aliphatic index for all the epitopes suggests the aliphatic side chain stable groups favor the thermostable nature of the peptides. The server also provided details on the number of atoms for the four peptides as 211, 166, 180, and 150, respectively. Findings on the extinction coefficient data had shown that there are no trypsin, tyrosine, or cysteine amino acids in the predicted proteins; thus, the proteins could not be visualized under UV spectrophotometry (server data).

Optimization of the drug delivery systems is known to be best achieved by analyzing the signal localization of the peptides, which could minimize the cost and labor, too. With the advent of servers like the SignalP latest version tool, it could be easily achieved as it predicts the proteins based on the transmembrane (TM) and no-TM neural networks and likelihood categorization for signal peptides through Signal 5.0 versions. The present finding did not include the peptides as TM proteins, though the UNIPROT database has documented both bfmR and bfmS as transmembrane proteins, which is a fact to be considered in the selection of the peptides as vaccine candidates. It is also a known fact that the peptide signals in the cytoplasmic membrane could be employed as small-molecule drug delivery systems.

Computational assessment for the selection of the T-cell dominant peptides from bfmR and bfmS was best achieved by IEDB T-cell prediction tools. The first two lowest percentile ranks, together with the consensus CombLib, SMM, NetPan, and ANN scoring and ranking for the IC50 values (<200 nm), were considered for the final selection of the T-cell dominant peptides. We selected the frequent HLA alleles present in at least 1% of the human population from the default tool for analyzing the HLA class I interactions with the selected peptide, which was so promising, and observed the same under the MHC cluster server as heat maps and graphical trees for the strength of the interactions. Final validation on the protein-peptide complexes with the TLR-2 receptor was also successfully achieved on GALAXY web servers, and it was so exciting to note that the peptides could interact with the receptors with high interaction similarity scores and hydrogen bonds. The promising epitopes, as predicted for bfmR and bfmS in the present study, suggest their potent role as possible candidates for the design of vaccines targeting the TCS of *A. baumannii*, recommending further in vitro and in vivo experimental validation.

Limitations

With the promising prediction of the peptides from the TCS components of A. baumannii, the objective of the study was not to evaluate the immunological response and memory through preclinical trials and would progress in the near future.

## Conclusions

To conclude, using the computational approach based on the reverse vaccinology technique, a first-of-its-kind investigation was undertaken for the epitope predictions from bfmR and bfmS of *A. baumannii*. The study suggests the possibility of promising vaccine design and evaluation targeting bfmR and bfmS to combat the TCS-mediated adaptations in *A. baumannii*. From the present investigation, potent epitopes that have been successfully elucidated using bioinformatics tools and databases may be considered for vaccine constructs in the near future. However, the study requires further in-vitro and preclinical assays for a better understanding of the immunological response and memory in the host biological environment.
